# *UBTF* tandem duplications are rare but recurrent alterations in adult AML and associated with younger age, myelodysplasia, and inferior outcome

**DOI:** 10.1038/s41408-023-00858-y

**Published:** 2023-05-26

**Authors:** Julia-Annabell Georgi, Sebastian Stasik, Jan-Niklas Eckardt, Sven Zukunft, Marita Hartwig, Christoph Röllig, Jan Moritz Middeke, Uta Oelschlägel, Utz Krug, Tim Sauer, Sebastian Scholl, Andreas Hochhaus, Tim H. Brümmendorf, Ralph Naumann, Björn Steffen, Hermann Einsele, Markus Schaich, Andreas Burchert, Andreas Neubauer, Kerstin Schäfer-Eckart, Christoph Schliemann, Stefan W. Krause, Mathias Hänel, Richard Noppeney, Ulrich Kaiser, Claudia D. Baldus, Martin Kaufmann, Carsten Müller-Tidow, Uwe Platzbecker, Wolfgang E. Berdel, Hubert Serve, Gerhard Ehninger, Martin Bornhäuser, Johannes Schetelig, Frank Kroschinsky, Christian Thiede, Study Alliance Leukemia (SAL)

**Affiliations:** 1 Medizinische Klinik und Poliklinik 1, Universitätsklinikum Carl Gustav Carus, Dresden, Germany; 2 Medizinische Klinik 3, Klinikum Leverkusen, Leverkusen, Germany; 3 Universität Heidelberg, Medizinische Klinik und Poliklinik, Abteilung Innere Medizin V, Heidelberg, Germany; 4 Klinik für Innere Medizin II, Universitätsklinikum Jena, Jena, Germany; 5 Medizinische Klinik IV, Uniklinik RWTH Aachen, Aachen, Germany; 6 Medizinische Klinik III, St. Marien-Krankenhaus Siegen, Siegen, Germany; 7 Medizinische Klinik 2, Hämatologie/Onkologie, Johann Wolfgang Goethe-Universität, Frankfurt am Main, Germany; 8 Medizinische Klinik und Poliklinik II, Universitätsklinikum Würzburg, Würzburg, Germany; 9 Klinik für Hämatologie, Onkologie und Palliativmedizin, Rems-Murr-Klinikum Winnenden, Winnenden, Germany; 10 Klinik für Innere Medizin, Schwerpunkt Hämatologie, Onkologie und Immunologie, Philipps Universität Marburg, Marburg, Germany; 11 Klinikum Nürnberg, Paracelsus Medizinische Privatuniversität, Medizinische Klinik 5, Nürnberg, Germany; 12 Medizinische Klinik A, Universitätsklinikum Münster, Münster, Germany; 13 Medizinische Klinik 5, Universitätsklinikum Erlangen, Erlangen, Germany; 14 Klinik für Innere Medizin III, Klinikum Chemnitz, Chemnitz, Germany; 15 Klinik für Hämatologie, Universitätsklinikum Essen, Essen, Germany; 16 Medizinische Klinik II, St. Bernward Krankenhaus, Hildesheim, Germany; 17 Klinik für Innere Medizin II, Universitätsklinikum Schleswig-Holstein, Campus Kiel, Kiel, Germany; 18 Abteilung für Hämatologie, Onkologie und Palliativmedizin, Robert-Bosch-Krankenhaus, Stuttgart, Germany; 19 Klinik und Poliklinik für Hämatologie, Zelltherapie und Hämostaseologie, Universitätsklinikum Leipzig, Leipzig, Germany; 20 AvenCell Europe GmbH, Dresden, Germany; 21 National Center for Tumor Diseases NCT, Dresden, Germany; 22 DKMS Clinical Trials Unit, Dresden, Germany; 23 AgenDix GmbH, Dresden, Germany

**Keywords:** Genetics research, Acute myeloid leukaemia

## Abstract

Tandem-duplication mutations of the *UBTF* gene (*UBTF*-TDs) coding for the upstream binding transcription factor have recently been described in pediatric patients with acute myeloid leukemia (AML) and were found to be associated with particular genetics (trisomy 8 (+8), *FLT3*-internal tandem duplications (*FLT3*-ITD), *WT1*-mutations) and inferior outcome. Due to limited knowledge on *UBTF-*TDs in adult AML, we screened 4247 newly diagnosed adult AML and higher-risk myelodysplastic syndrome (MDS) patients using high-resolution fragment analysis. *UBTF*-TDs were overall rare (*n* = 52/4247; 1.2%), but significantly enriched in younger patients (median age 41 years) and associated with MDS-related morphology as well as significantly lower hemoglobin and platelet levels. Patients with *UBTF*-TDs had significantly higher rates of +8 (34% vs. 9%), *WT1* (52% vs. 7%) and *FLT3*-ITD (50% vs. 20.8%) co-mutations, whereas *UBTF*-TDs were mutually exclusive with several class-defining lesions such as mutant *NPM1*, in-frame *CEBPA*^bZIP^ mutations as well as t(8;21). Based on the high-variant allele frequency found and the fact that all relapsed patients analyzed (*n* = 5) retained the *UBTF*-TD mutation, *UBTF*-TDs represent early clonal events and are stable over the disease course. In univariate analysis, *UBTF*-TDs did not represent a significant factor for overall or relapse-free survival in the entire cohort. However, in patients under 50 years of age, who represent the majority of *UBTF*-mutant patients, *UBTF*-TDs were an independent prognostic factor for inferior event-free (EFS), relapse-free (RFS) and overall survival (OS), which was confirmed by multivariable analyses including established risk factors such as age and ELN2022 genetic risk groups (EFS [HR: 2.20; 95% CI 1.52–3.17, *p* < 0.001], RFS [HR: 1.59; 95% CI 1.02–2.46, *p* = 0.039] and OS [HR: 1.64; 95% CI 1.08–2.49, *p* = 0.020]). In summary, *UBTF*-TDs appear to represent a novel class-defining lesion not only in pediatric AML but also younger adults and are associated with myelodysplasia and inferior outcome in these patients.

## Introduction

The upstream binding transcription factor (UBF or UBTF), encoded by the *UBTF* gene located on chromosome 17, is a member of the high mobility group (HMG)‑box protein family, a group of ubiquitously expressed non-histone architectural proteins (reviewed in ref. [1]). UBTF is a key regulator of ribosomal RNA transcription, mediating the recruitment of RNA polymerase I to rDNA promoter regions through the formation of nucleosome free regions [2] and the assembly of the preinitiation complex [3, 4]. UBTF is expressed in two variant isoforms as the result of differential splicing, with the shorter variant UBTF2 lacking a segment of HMG-Box 2 [5]. Recent data suggest that the UBTF1/2 ratio regulates the rate of rRNA synthesis and determines the sensitivity of rRNA genes to growth factor stimulation in different cell types [6]. Both Germline and somatic genomic aberrations of *UBTF* have been linked to several diseases, including childhood neurodegenerative disorders [7, 8], solid tumors such as melanoma [9] or colorectal cancer [10], and hematopoietic malignancies such as precursor acute lymphoblastic leukemia [11]. Recently, tandem duplications in exon 13 of *UBTF* (*UBTF*-TDs) were identified as novel, recurrent alterations in newly diagnosed and relapsed cases of pediatric acute myeloid leukemia (AML) by a whole genome sequencing approach [12–14]. In children, *UBTF*-TDs were associated with poor outcome and a distinct genetic profile, including high rates of trisomy 8 (+8), *FLT3*-internal tandem duplications (*FLT3*-ITDs) and *WT1*-mutations. To date, data on the role of *UBTF*-TDs in adults are limited, prompting us to investigate the prevalence and prognostic impact of *UBTF-*TDs in a large and well characterized cohort of adult AML patients.

## Patients, materials, and methods

### Patients

A total of 4247 adult patients with newly diagnosed AML (*n* = 3300 de novo AML, *n* = 796 secondary or therapy-related AML) or higher-risk myelodysplastic syndrome (HR-MDS, *n* = 151) were retrospectively screened for the presence of *UBTF*-TDs. Patients with appropriate genetic data (*n* = 1456) were re-stratified according to ELN2022 [15] and included in the outcome analyses. Most of these patients (*n* = 1265) were treated in prospective studies involving risk stratified post induction therapy according to cytogenetic risk groups, i. e. the AML96, AML2003, AML60+ and SORAML protocols of the Study Alliance Leukemia (SAL); the remaining patients (*n* = 191) were recruited to the SAL registry. Detailed treatment protocols have been published previously [16–19] and are summarized in the supplement, including the number of patients treated in each protocol (Table S1). This study was approved by the ethical board of the Medical Faculty TU Dresden. Each patient gave written informed consent to participate in the respective studies.

### Patient samples

All materials investigated were obtained at the time of diagnosis. Bone marrow was used whenever available, in all other cases, peripheral blood samples were examined. Genomic DNA was extracted from mononuclear cells using standard procedures (DNA Blood mini kit, Qiagen, Hilden, Germany).

### Mutational analysis of *UBTF*

*UBTF*-TD screening was done by PCR covering exon 13 of the *UBTF* gene with 6-FAM labeled primers followed by high-resolution fragment analysis. PCR amplified mutant samples were purified and sequenced on an ABI3130xl instrument (Life Technologies, Darmstadt, Germany). Details of the PCR primers and cycling conditions are given in the supplement (Table S2 and S3).

### Next generation sequencing (NGS)-based characterization of co-mutations in *UBTF* mutant patients

Profiling of mutations in *UBTF* mutant samples was done by targeted NGS-based resequencing using the Archer VariantPlex Myeloid panel (Illumina, Chesterford, UK) covering 75 genes frequently mutated in AML as described recently [20]. Samples were sequenced paired-end (150 bp PE) on NextSeq- (Illumina) or (300 bp PE) MiSeq-NGS platforms. Sequence data alignment of demultiplexed FastQ files, variant calling and filtering was done using the Sequence Pilot software package (JSI medical systems GmbH, Ettenheim, Germany) with default settings and a 5% variant allele frequency (VAF) mutation calling cut-off.

### RNA-sequencing

RNA sequencing was performed on total RNA isolated at diagnosis from 7 AML samples from *UBTF*-TD^pos^ patients and 42 samples from other well established AML subgroups (t(8;21), t(6;9); inv16, *NPM1* and in-frame *CEBPA*^bZIP^ mutant patients, and patients with a *NUP98-NSD1* fusion) using strand-specific RNA-Seq library preparation (Ultra II Directional RNA Library Prep, NEB) and sequenced on an Illumina NovaSeq 6000 instrument. The complete workflow as well as the bioinformatic analysis are detailed in the supplement.

### Statistical analysis

All statistical analyses were performed using R version 4.2.0 (https://www.R-project.org/.) and STATA BE 17.0 (Stata Corp, College Station, TX, USA). All analyses were carried out as two‑sided tests. Statistical significance was determined using a significance level α of 0.05. Clinical variables across groups were compared using the Fisher’s exact test for categorial variables, the nonparametric Mann-Whitney U test was applied for continuous variables. *p*-values of association analyses of *UBTF* mutations with clinical variables and other molecular abnormalities were adjusted for multiplicity using the Bonferroni-Holm-procedure. With regard to outcome analysis, univariate analysis was carried out using logistic regression to obtain odds ratios (OR). Time-to-event analysis was performed using Cox-proportional hazard models to obtain hazard ratios (HR) as well as the Kaplan–Meier method and the log-rank-test. Multivariable models were adjusted for ELN2022 risk categories [15] and age. Median follow-up time was calculated using the reverse Kaplan–Meier method.

## Results

**Fig. 1:**
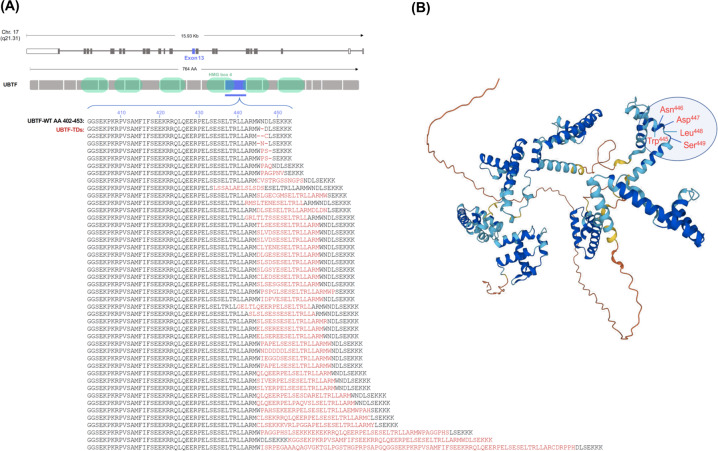
Illustration of the effect of *UBTF* tandem duplications on the amino acid level. **A** All patients showed typical inframe insertions and/or deletions leading to alterations in the coding sequence of exon 13. **B** 3D protein structure of UBTF (Source: Alphafold.ebi.ac.uk Sequence AF-P17480-F1). The highlighted amino acids represent the localization hotspots AA 445-449 of the observed InDel mutations.

**Fig. 2:**
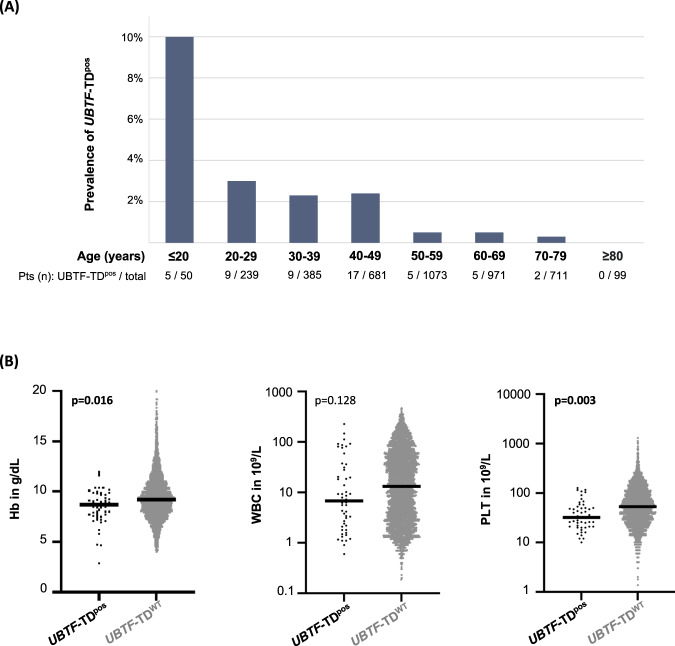
Clinical variables of *UBTF*-TD^pos^ patients. **A** Age distribution of the 52 *UBTF*-TD^pos^ patients. Prevalence of *UBTF*-TDs shows a strong negative correlation with age. **B** Peripheral blood counts in *UBTF*-TD^pos^ vs. *UBTF*-TD^WT^ patients. Despite their young age, laboratory parameters revealed significantly lower hemoglobin levels and platelet counts at diagnosis for *UBTF*-TD^pos^ patients.

*UBTF-*TDs were identified in 52 (1.2%) of 4247 patients analyzed. All mutations yielded *in-frame* insertions, duplications and/or deletions in *UBTF* exon 13 (median length 51 base pairs; range –6 to +312 bp), specifically affecting the link between the second and third a-helix of HMG-box 4 (Fig. 1). Although *UBTF*-TDs were rare across the overall cohort, they were considerably more common in younger patients and showed an inverse correlation with age, ranging from 10% in patients below 20 years to 0.25% in patients over 70 years (Fig. 2). Accordingly, the median age was significantly lower in patients with *UBTF*-TDs (41 years; IQR 28–48.5 years) than in *UBTF*-TD^WT^ patients (57 years; IQR 46–67 years; *p* < .001). Based on local evaluation, a high percentage of cases were initially classified as AML M6 at diagnosis, that would qualify as MDS since 2016 [21]. Central cytomorphologic re-assessment of available slides (*n* = 41) in *UBTF*-TD^pos^ patients confirmed a strong association with myelodysplastic changes (*n* = 25/41; 61%; Fig. S1), supporting data in pediatric MDS [22], where *UBTF*-TDs were found in 25% of patients. This might indicate that in *UBTF* mutant AML, the disease frequently evolves from preexisting MDS. In line with this, laboratory parameters revealed significantly lower hemoglobin levels and platelet counts at diagnosis for *UBTF*-TD^pos^ patients compared to *UBTF*-TD^WT^ patients (median Hb 8.7 vs. 9.2 g/dL; *p* = 0.02; median PLT 31.5 vs. 53 × 10^9^/L; *p* = 0.003; Table 1, Fig. 2).

**Table 1:** Clinical and genetic variables in *UBTF*-TD^pos^ and *UBTF*-TD^WT^ patients.

	*UBTF-*TD^WT^ *n* = 4195	*UBTF-*TD^pos^ *n* = 52	*p*-value (adj.)
Age, years, median (IQR)	57 (46–67)	41 (28–48.5)	**<0.001**
Sex, *n* (%)			
Female	2038 (49)	22 (42)	0.368
Male	2157 (51)	30 (58)	
AML type, *n* (%)			
de novo	3261 (78)	39 (75)	0.080
sAML	548 (13)	11 (22)	
tAML	237 (6)	-	
HR-MDS	149 (3)	2 (3)	
Laboratory, median (IQR)			
BM blasts, %	61 (38–80)	46.5 (30.5–71)	0.057
WBC, 10^9^/L	13.3 (3–41.6)	6.3 (1.7–20)	0.128
PLT, 10^9^/L	53 (25–98)	32 (21–50)	**0.003**
Hb, g/dL	9.2 (8.0–10.6)	8.7 (7.5–9.6)	**0.016**
FAB subtype, *n* (%)		-	
M0	446 (11)	4 (8)	**<0.001**
M1	846 (21)	8 (15)	
M2	1143 (28)	14 (27)	
M4	702 (17)	6 (12)	
M5	559 (14)	4 (8)	
M6	117 (3)	12 (23)	
M7	27 (1)	1 (2)	
RAEB	88 (2)	2 (3)	
RAEB-T	138 (3)	1 (2)	
n-miss	129	-	
Cytogenetic risk ELN 2022, *n* (%)			
Favorable	322 (8)	-	0.070
Intermediate	2802 (70)	47 (90)	
Adverse	902 (22)	3 (10)	
n-miss	169	2	
Trisomy 8	372 (9)	19 (36.5)	**<0.001**
*NPM1*^mut^, *n* (%)	1176 (28)	-	**<0.001**
*FLT3-*ITD^pos^, *n* (%)	872 (20.8)	26 (50)	**<0.001**
*CEBPA*^bZIP-inf^, *n* (%)	157 (3.7)	-	**<0.001**

*IQR* interquartile range, *AML* acute myeloid leukemia, *sAML* secondary AML, *tAML* therapy-related AML, *HR-MDS* higher-risk myelodysplastic syndrome, *BM* bone marrow, *WBC* white blood cell count, *PLT* platelet counts, *Hb* hemoglobin levels, *FAB* French-American-British, *RAEB* refractory anemia with excess blasts, *RAEB-T* refractory anemia with excess blasts in transformation, *ELN* European Leukemia Network, *CR* complete remission, *OS* overall survival, *RFS* relapse-free survival, *alloHSCT* allogeneic hematopoietic stem cell transplantation.

Bold values indicates statisically significant *p* values.

**Fig. 3:**
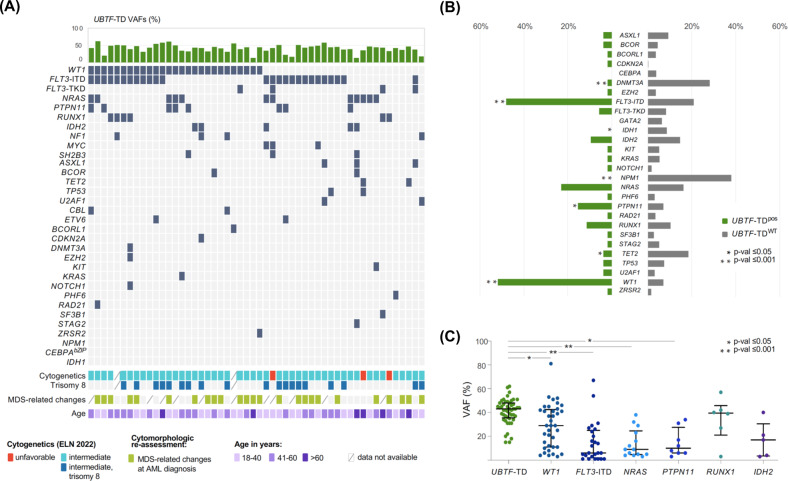
Landscape of co-mutations in *UBTF* mutant AML and survival analysis according to *UBTF* mutant status. **A** Alignment of additional gene mutations in 52 *UBTF*-TD^pos^ patients. **B** Frequency distribution of additional gene mutations identified in *UBTF*-TD^pos^ vs. *UBTF*-TD^WT^ patients. **C** Variant allele frequencies (VAFs) of *UBTF*-TDs and frequent co-mutations (frequency of at least 10% in *UBTF*-mut patients). Solid bars indicate VAF median and IQR.

As in pediatric patients [13, 14], there was a significant association with +8 (38% in *UBTF*-TD^pos^ vs. 9.8% *UBTF*-TD^WT^; *p* < 0.001), 3 patients (6%) showed an adverse karyotype. Similar to findings in children, *UBTF*-TDs were significantly associated with *WT1*-mutations (52% vs. 8.6, *p* < 0.001), *FLT3*-ITDs (50% vs. 20.8%, *p* < 0.001) as well as *PTPN11*-mutations (15.4% vs. 7%; *p* = 0.022), while *DNMT3A* (1.9% vs. 28.1%; *p* < 0.001), *TET2* (3.8% vs. 18.4%; *p* = 0.007) and *IDH1*-mutations (0% vs. 8.6%; *p* = 0.027) were significantly less common (Fig. 3A/B, Table S4). Furthermore, *UBTF*-TDs and several class-defining lesions [23], i.e., reciprocal translocations such as t(8;21), t(6;9) or mutations in *CEBPA*^bZIP^ or *NPM1*, were mutually exclusive (Fig. 3A/B). As outlined in Fig. 3C, the variant allele frequency (VAF) of *UBTF*-TDs (median 43%; range 13-62%) was significantly higher than the VAFs of frequent co-mutations, indicating that the *UBTF*-TD may represented the earliest clonal event.

In order to further assess the persistence of *UBTF*-TDs in patients in complete morphologic remission (CR) we screened DNA from available remission samples in 9 patients using the fragment analysis procedure, which has a sensitivity of about 1%. In 5 of these 9 samples, residual *UBTF*-TD mutations were still detectable (median VAF 2.7%; range 0.9–4.3%), which is in line with the overall rather low CR rate of 58.8% for these patients.

Relapse samples were available in five patients. In all patients, the initially detected *UBTF*-TD mutation was also present at the time of relapse. In contrast, the comutational profile showed evidence of profound changes with losses of mutations in *NRAS, PTPN11* and *FLT3* and acquisition of mutations in *NF1, BCOR, WT1, GATA2* and *U2AF1* (Fig. S2).

**Fig. 4:**
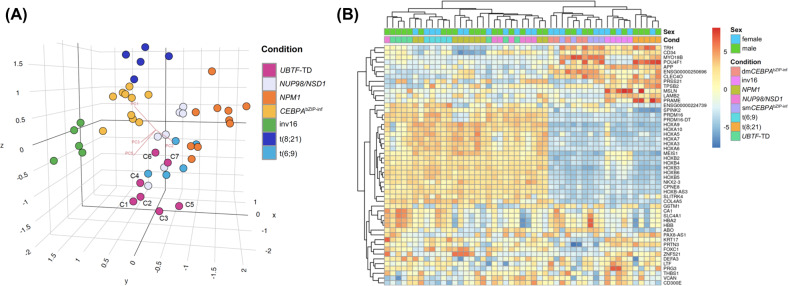
RNA-sequencing analysis in *UBTF* mutant AML and other well established AML subgroups. **A** Principal component analysis (PCA) of the RNA-Sequencing data. **B** Heat map of RNA-Sequencing analysis indicates the top 50 differentially expressed genes ranked based on FDR between *UBTF*-TD^pos^ patients and references, with high levels of expression shown in red and low levels shown in blue. Color coding is based on standardized and normalized read counts accounting for the library size.

To gain additional insights into the biology of *UBTF*-TD mutant AML, we performed an RNA-sequencing analysis of 7 AML samples from *UBTF*-TD^pos^ patients and 42 samples from other well established AML subgroups, i.e., t(8;21), inv16, *NPM1* and in-frame *CEBPA*^bZIP^ mutant patients (single and double), t(6;9) and patients with a *NUP98-NSD1* fusion. As shown in Fig. 4A, the principle component analysis (PCA) revealed a clustering of the *UBTF*-TD^pos^ samples which showed a partial overlap with those of *NUP98-NSD1* patients and patients with t(6;9), which is consistent with previous results [13]. A heatmap built on unsupervised clustering based on the top 50 differentially expressed genes showed two major clusters containing the *UBTF*-TD^pos^ samples, one of which again showed an overlap between the *UBTF*-TDs and *NUP98-NSD1* and t(6;9) patients (Fig. 4B). In line with previous reports [13, 14], *UBTF*-TD^pos^ samples showed a strong upregulation of several HOX-genes (*HOX-A* and *HOX-B*; Fig. 4B, Fig. S3). Interestingly, four of the seven *UBTF*-TD^pos^ samples showed a strong upregulation of several genes associated with erythroid differentiation (*HBA2, HBB, ABO*), which might explain the association with the erythroid lineage/FAB M6. Pairwise comparison between *UBTF*-TD and the other subgroups for the most differentially expressed genes again showed the smallest difference compared with patients with t(6;9) (396 differentially expressed genes (DEGs)) and *NUP98-NSD1* fusion (455 DEGs), whereas the largest differences were observed compared with patients with in-frame mutations in *CEBPA*^bZIP^ (1278 DEGs) and t(8;21) (1424 DEGs) (Fig. S3).

**Fig. 5:**
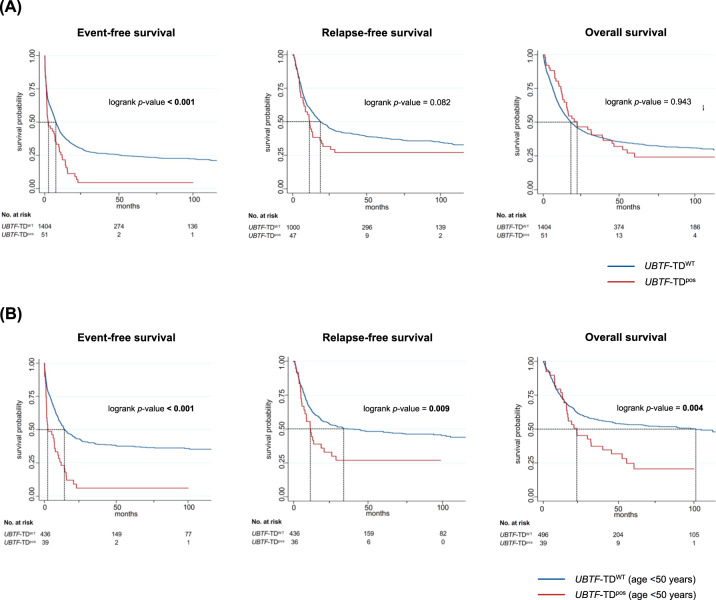
Survival analysis according to *UBTF* mutant status. Kaplan–Meier survival curves showing event-free, relapse-free and overall survival of *UBTF*-TD^pos^ and *UBTF*-TD^WT^ patients for **A** all patients and **B** for patients <50 years of age. *p*-values were calculated using the log-rank test. Numbers of patients at risk are provided below the *x*-axis.

The prognostic relevance of *UBTF-*TD mutations was analyzed in 1455 intensively treated patients, which had available NGS data allowing to reclassify them according to the ELN2022 risk groups (1404 *UBTF-*TD^WT^/51 *UBTF-*TD^pos^; median follow-up time of patients alive 61 months; IQR 36–96 months). Individual clinical courses of all *UBTF*-TD^pos^ patients are summarized in Table S5. In the entire cohort, *UBTF*-TDs were associated with a significantly shorter event-free survival (EFS, median *UBTF*-TD^pos^ 2.4 months vs. *UBTF*-TD^WT^ 7.5 months; *p* < 0.001), no differences in relapse-free survival (RFS) or overall survival (OS) were observed (Fig. 5A). In a subgroup analysis in patients <50 years of age, who represent the majority of *UBTF* mutant patients (40/52 = 77%; 3% *UBTF*-TD prevalence in pts <50 years vs. 0.4% in pts ≥50 years), *UBTF*-TDs were associated with significantly shorter EFS (median EFS *UBTF*-TD^pos^ 2.4 [1.5–7.7] months vs. *UBTF*-TD^WT^ 14.0 [11.6–19.3] months; *p* < 0.001), RFS (median RFS *UBTF*-TD^pos^ 11.2 [5.6-20.4] months vs. *UBTF*-TD^WT^ 35.9 [23.0-102.4] months; *p* = 0.009) and OS (median OS *UBTF*-TD^pos^ 22.3 [15.4–39.5] months vs. *UBTF*-TD^WT^ 101.1 [46.9-n.r.] months; *p* = 0.005) (Fig. 5B). Multivariable analyses (including age and ELN2022 risk group), confirmed *UBTF*-TDs as an independent risk factor for dismal EFS (HR: 2.20; 95% CI 1.52–3.17, *p* < 0.001), RFS (HR: 1.59; 95% CI 1.02–2.46, *p* = 0.039) and OS (HR: 1.64; 95% CI 1.08–2.49, *p* = 0.020) in patients <50 years of age (Table S6). The lack of this effect in the overall cohort (Table S7) can probably be explained by the generally poorer outcome of AML patients at higher ages and the low prevalence of *UBTF*-TD mutations in older patients. To address whether the observed survival differences in younger adults merely result from the absence of favorable risk features such as *NPM1* or in-frame *CEBPA*^bZIP^ mutations, which appear to be mutually exclusive with the presence of *UBTF*-TDs, we performed an additional subgroup analysis in patients stratified as intermediate risk according to ELN2022 guidelines [15]. Although also in these patients *UBTF*-TDs were associated with an inferior overall (median OS *UBTF*-TD^pos^ 22.4 [15.8-55.4] months vs. *UBTF*-TD^WT^ 97.7 [20.6-n.r.] months; *p* = 0.204) and relapse-free survival (median RFS *UBTF*-TD^pos^ 11.5 [5.6–25.6] months vs. *UBTF*-TD^WT^ 27.9 [12.1-n.r.] months; *p* = 0.189), only the event-free survival showed a significant difference between *UBTF*-TD^pos^ and *UBTF*-TD^WT^ patients (median EFS *UBTF*-TD^pos^ 2.1 [1.4-7.7] months vs. *UBTF*-TD^WT^ 11.1 [7.8-24.5] months; *p* < .001), presumably due to the limited number of patients in this analysis (Fig. S4). For the two most common co-mutations, *FLT3*-ITD and *WT1*, there was no evidence of a relevant additional effect on outcome (Fig. S5A). Clearly, these data have to be interpreted with caution, given these even smaller subsets of patients.

Allogeneic hematopoietic stem cell transplantation (alloHSCT) performed in CR1 in 11 patients (22% vs. 15% in *UBTF*-TD^WT^ patients) did not improve outcome in this limited data set (Fig. S6), but alloHSCT performed as salvage treatment (30/51 patients, 59%) was the only intervention associated with long-term cure, thus the impact of alloHSCT on outcome cannot be finally addressed in this study due to small numbers.

## Discussion

Summarizing these results, *UBTF*-TDs appear to characterize a novel class-defining lesion not only in children but also younger adult AML patients. In vitro studies confirmed that *UBTF*-TDs confer a proliferative advantage to cord-blood derived CD34+-cells [13]. However, the functional implications of these mutations are unknown. All *UBTF*-TDs reported so far are *in-frame* alterations and affect the HMG-box 4 of UBTF, indicating that these changes are not loss- but gain- or shift-of-function lesions. Among the 6 HMG-boxes of UBTF, HMG-box 4 is unique to mammalian UBTF and appears to regulate species specificity [24]. Since the UBTF-wt protein is predominantly located in the nucleolus and interacts with numerous other nucleosomal proteins, including NPM1, an important regulator of the p14^ARF^/HDM2/TP53 axis [25], one might speculate that altered protein binding of mutant UBTF and sequestration of other essential proteins are involved in the transformation process.

An interesting observation not previously described is the intriguing association of *UBTF*-mutations with myelodysplastic features seen in our cohort. This observation and the particularly high rate of *UBTF*-TDs in pediatric MDS patients [22] together point to the fact that the leukemia in these patients might originate from a prior MDS clone. The high association with the former AML M6 subtype also suggests a defect in erythroid differentiation. Interestingly, a recent comprehensive molecular study of erythroleukemia patients reported 3 patients with 6 bp-deletions in *UBTF*, similar to those which we observed in our cohort [26].

Transcriptomic profiling via RNA-sequencing revealed several interesting aspects of *UBTF*-TD mutant AML. As reported previously, samples from *UBTF*-TD^pos^ patients clustered together with *NUP98/NSD1*, t(6;9) and *NPM1*^mut^. All these subgroups show a strong upregulation of HOX-genes, most importantly *HOXA* as well *HOXB*. Another gene highly upregulated in this subgroup is *PRDM16*. *PRDM16* codes for a histone H3K4 methyltransferase which is involved in adipose tissue differentiation, neural stem cell maintenance and represents an important regulator of normal hematopoietic differentiation [27]. In mouse models, forced overexpression of murine PRDM16, especially the short isoform, is able to transform hematopoietic stem cells and induce a fatal, transplantable leukemia [28]. In human AML, overexpression of *PRDM16* has been linked to *NUP98-NSD1* fusions as well as *NPM1* mutant AML and was found to be associated with poor prognosis [29]. Interestingly and in line with the observed association with AML M6 discussed above, samples from *UBTF*-TD patients also showed an upregulation of several genes associated with erythroid differentiation, e.g., *HBB, HBA2*, and *ABO*.

The poor prognosis found in our study as well as in the pediatric patients, despite treatment with alloHSCT, indicates that novel treatment algorithms need to be evaluated in these patients. Interestingly, overexpression of *HOXA* and *HOXB* genes has recently been described as a predictive biomarker for sensitivity to treatment with BCL2-inhibitors such as Venetoclax [30], which is in line with the favorable response to Venetoclax treatment observed especially in *NPM1* mutant patients [31]. However, more recent data suggest that in patients with erythroid differentiation, the BCX-L pathway might be more relevant, which would suggest that other inhibitors, such as Navitoclax [32] should be taken into account.

Taken together, these results as well as previously published data indicate that *UBTF*-TDs characterize a novel and specific subtype of AML, predominantly affecting adolescents and younger adults. The fact that *UBTF*-TDs appear to represent early clonal lesions, which have a very specific pattern of cytogenetic alterations (+8) and co-mutations (*FLT3*-ITD and *WT1*) as well as the absence of other subtype-specific lesions such as mutations in *NPM1*, *CEBPA*^bZIP^ or CBF-translocations, point to *UBTF*-TD as a novel class-defining lesion in AML. Based on the significantly higher prevalence in children and adolescents as well as the fact that the gene is not covered in most clinically used diagnostic assays, dedicated screening for this mutation should be considered in patients below the age of 50 years. Due to the overall poor response to chemotherapy and alloHSCT, further understanding of the causative mechanisms appears crucial to improve the treatment and prognosis of these patients.

## Supplementary information

Supplement

## Data Availability

The datasets generated and/or analyzed during the current study are available in the Kaggle repository, 10.34740/kaggle/dsv/5550865.
