# Supplementary material for: UBTF tandem duplications are rare but recurrent alterations in adult AML and associated with younger age, myelodysplasia, and inferior outcome
Source: Blood Cancer J. 2023 May 26;13(1):88. doi: 10.1038/s41408-023-00858-y (PMC10220021; doi:10.1038/s41408-023-00858-y)

Supplementary Data

Supplementary Methods

UBTF analysis and Next Generation Sequencing (NGS)

All mutational studies were performed on DNA from bone marrow aspirates or peripheral blood taken at diagnosis. DNA was extracted using the DNeasy blood and tissue kit (Qiagen, Hilden, Germany) and quantified with the NanoDrop spectrophotometer. PCR for *UBTF* Exon 13-fragment analysis was performed using the following conditions: 20 ng of DNA were amplified in a volume of 30 µl containing 3 µl 10X TBE buffer, primers (10 µM, 1.5 µl of each), 2.4 µl dNTPs, 1.45 µl Aqua dest and 0.15 µl AmpliTaq Gold polymerase. High resolution capillary electrophoresis was performed on automated sequencers (ABI3130xl and ABI310 DNA; Life Technologies, Darmstadt, Germany).

Profiling of co-mutations was done by targeted resequencing using the TruSight Myeloid panel (Illumina, Chesterford, UK) for *UBTF*-TD^{WT} patients and Archer VariantPlex Myeloid panel (Illumina, Chesterford, UK) for *UBTF*-TD^{pos} patients.

For each reaction, 50 ng of genomic DNA was used. Library preparation was done as recommended by the manufacturer. Samples were sequenced paired-end (150 bp PE) on NextSeq- (Illumina) or (300 bp PE) MiSeq-NGS platforms. Sequence data alignment of demultiplexed FastQ files, variant calling and filtering was done using the Sequence Pilot software package (JSI medical systems GmbH, Ettenheim, Germany) with default settings and a 5% variant allele frequency (VAF) mutation calling cut-off. Human genome build HG19 was used as reference genome for mapping algorithms.

RNA-Sequencing

mRNA was isolated from 300ng total RNA by poly-dT enrichment using the NEBNext Poly(A) mRNA Magnetic Isolation Module (NEB) according to the manufacturer's instructions. Samples were then directly subjected to the workflow for strand-specific RNA-Seq library preparation (Ultra II Directional RNA Library Prep, NEB). For ligation custom adaptors were used. After ligation, adapters were depleted by an XP bead purification (Beckman Coulter) adding the beads solution in a ratio of 1:0.9. Dual unique indexing was done during the following PCR enrichment (12 cycles, 65 °C) using custom amplification primers carrying the same sequence for i7 and i5 index. After two more XP bead purifications (1:0.9), libraries were quantified using the Fragment Analyzer (Agilent). Libraries were sequenced as 2 x 100 bp on an Illumina NovaSeq 6000 with an average of 50 million fragments per library.

After sequencing, FastQC (http://www.bioinformatics.babraham.ac.uk/) was used to perform a basic quality control on the resulting sequencing data. Fragments were then aligned to the human reference genome hg38 with the support of the Ensembl 98 transcriptome annotation and the aligner gsnap (v2020-12-16) [1], [2]. The table of fragments per gene was created based on the overlap of the uniquely mapped fragments with the same Ensembl annotation using featureCounts (v2.0.1) [3]. Normalization of raw fragments based on the library size and testing for differential expression between the different cell types/treatments was performed with the DESeq R package (v1.30.1) [4]. Sample to sample Euclidean distance, Pearson' and Spearman correlation coefficient (r) and PCA based upon the top 500 genes showing highest variance were computed to explore correlation between biological replicates and different libraries. To identify differentially expressed genes, counts were fitted to the negative binomial distribution and genes were tested between conditions using the Wald test of DESeq2 including sex as a covariate. Resulting p-values were corrected for multiple testing with the Independent Hypothesis Weighting package (IHW 1.12.0) [5], [6]. Genes with a maximum of 5% false discovery rate (p-adj ≤ 0.05) were considered as significantly differentially expressed.

References

[1] Thomas D. Wu and Colin K. Watanabe. “GMAP: a genomic mapping and alignment program for mRNA and EST sequences”. eng. In: Bioinformatics (Oxford, England) 21.9 (May 2005), pp. 1859–1875. issn: 1367-4803. doi: 10.1093/bioinformatics/bti310.

[2] Thomas D. Wu and Serban Nacu. “Fast and SNP-tolerant detection of complex variants and splicing in short reads“ Bioinformatics 2010 26:873-881

[3] Yang Liao, Gordon K. Smyth, and Wei Shi. “featureCounts: an efficient general purpose program for assigning sequence reads to genomic features”. eng. In: Bioinformatics (Oxford, England) 30.7 (Apr. 2014), pp. 923–930. issn: 1367-4811. doi: 10.1093/bioinformatics/btt656.

[4] Love MI, Huber W, Anders S (2014). “Moderated estimation of fold change and dispersion for RNA-seq data with DESeq2.” Genome Biology, 15, 550. doi: 10.1186/s13059-014-0550-8.

[5] Ignatiadis N, Klaus B, Zaugg J, Huber W (2016). “Data-driven hypothesis weighting increases detection power in genome-scale multiple testing.” Nature Methods. doi: 10.1038/nmeth.3885.

[6] Ignatiadis N, Huber W (2017). “Covariate-powered weighted multiple testing with false discovery rate control.” arXiv. doi: arXiv:1701.05179.

Detailed description of clinical protocols

AML96 (ClinicalTrials.gov Identifier: NCT00180115) was a prospective, open-label, randomized trial designed to assess the feasibility of a risk-adapted post-remission treatment strategy for patients with acute myeloid leukemia (Schaich et al., 2011; PMID: 21606413). All patients under 60 years of age received double induction therapy consisting of one cycle mitoxantrone, cytarabine, etoposide followed by one cycle cytarabine, amsacrine. The post-remission therapy for patients who achieved complete remission was stratified according to the cytogenic risk profile. Allogeneic HSCT was performed in patients with poor- or intermediate-risk profile and a HLA-matched donor. Patients without an available donor were randomly assigned to receive either H-MAC or I-MAC as first post-remission therapy followed by subsequent autologous HSCT or an additional cycle of MAMAC as second post-remission therapy.

Patients over 60 years of age were treated with double induction therapy (daunorubicin, cytarabine) followed by consolidation therapy (intermediate-dose cytarabine, amsacrine). They were included in the **AML60+** study (ClinicalTrials.gov Identifier: NCT00180167), a randomized clinical trial comparison between the conventional DA 7+3 protocol for elderly patients and an induction treatment protocol with mitoxantron and intermediate-dose cytarabine (Röllig et al., 2018; PMID: 29390048).

AML2003 (ClinicalTrials.gov Identifier: NCT00180102) was a prospective, randomized, open-label trial investigating the value of an intensified transplantation strategy and multiagent consolidation therapy for patients under the age of 60 years (Schetelig et al., 2015; PMID: 25434303). Patients were assigned into 4 treatment arms. In the arms A and C representing non-intensified transplantation strategy patients received double induction therapy with daunorubicin, cytarabine. Patients not harboring t(8;21) with an HLA-matching donor were scheduled for allogeneic HSCT in complete remission. Patients assigned to arms B and D received a risk-adapted post-remission therapy depending on cytogenetics, molecular biology and day15 BM results. In patients within the unfavorable risk group and with an HLA-matching donor, allogeneic HSCT was performed in aplasia after the first course of induction treatment. Intermediate-risk patients were scheduled for allogeneic HSCT in first complete remission after a second course of induction therapy. Patients without available donor were offered autologous HSCT. Patients who were not assigned for transplantation were randomized between post-remission chemotherapy consisting of either 3 x high-dose cytarabine (arms A and B) or MAC-MAMAC-MAC (arms C and D).

The **SORAML** trial (ClinicalTrials.gov Identifier: NCT00893373) evaluated the efficacy of sorafenib added to standard chemotherapy (Röllig et al., 2015; PMID: 26549589). Patients received double induction therapy with daunorubicin plus cytarabine followed by three cycles of high-dose cytarabine consolidation therapy plus either sorafenib or placebo on days 10–

19 of induction cycles, from day 8 of each consolidation, and as maintenance for 12 months. Allogeneic HSCT was scheduled for intermediate-risk patients with a sibling donor and for all high-risk patients with an HLA-matched donor in first remission.

Participating centers of the Study Alliance Leukemia (SAL)

Universitätsklinikum Aachen der RWTH, Aachen, Prof. Dr. Tim H. Brümmendorf; Klinikum Altenburger Land GmbH, Altenburg, Dr.med. Armin Schulz-Abelius; Klinikum Augsburg, Augsburg, Prof. Dr. Martin Trepel; Sozialstiftung Bamberg, Bamberg, Dr.med. Martina Teichmann; Klinikum Bayreuth GmbH, Bayreuth, Prof. Dr. Alexander Kiani; Helios Klinikum Berlin-Buch, Berlin, Prof. Dr. Bertram Glaß; Städt. Kliniken Bielefeld gGmbH, Bielefeld, PD Dr.med. Martin Görner; Augusta-Kranken-Anstalt gGmbH, Bochum, Prof. Dr. Dirk Behringer; Ev. Diakonie-Krankenhaus gGmbH, Bremen, Dr.med. Johannes Kullmer; Klinikum Chemnitz gGmbH, Chemnitz, PD Dr.med. Mathias Hänel; MVZ Coburg, Coburg, PD Dr.med. Christof Lamberti; Carl-Thiem-Klinikum Cottbus gGmbH, Cottbus, PD Dr.med. Martin Schmidt-Hieber; Universitätsklinikum Dresden, Dresden, Prof. Dr. Christoph Röllig; Krankenhaus Düren gGmbH, Düren, PD Dr.med. Michael Flaßhove; Universitätsklinikum Erlangen-Nürnberg, Erlangen, Prof. Dr. med. Andreas Mackensen; Universitätsklinikum Essen, Essen, Dr.med. Maher Hanoun; Klinikum Frankfurt (Oder) GmbH, Frankfurt (Oder), Prof. Dr. Michael Kiehl; Klinikum der J.W. Goethe Universität, Frankfurt (Main), Prof. Dr. Hubert Serve; Klinikum Fulda, Fulda, Prof. Dr. H.-G. Höffkes; Universitätsklinikum Halle (Saale), Halle (Saale), Dr.med. Lutz Peter Müller; St. Barbara-Klinik Hamm, Hamm, Dr. Dr. Heinz Albert Dürk; Universitätsklinikum Heidelberg, Heidelberg, Prof. Dr. med. Carsten Müller-Tidow; St. Bernward Krankenhaus, Hildesheim, Prof. Dr. Ulrich Kaiser; Universitätsklinikum Jena, Jena, Prof. Dr. Andreas Hochhaus; Westpfalz-Klinikum GmbH, Kaiserslautern, Prof. Dr. Gerhard Held; Städt. Krankenhaus Kiel, Kiel, Prof. Dr. Roland Peter Repp; Universitätsklinikum Schleswig-Holstein, Kiel, Prof. Dr. Claudia Baldus; Gemeinschaftsklinikum Mittelrhein gGmbH, Koblenz, Prof. Dr. Jens-Marcus Chemnitz; Universitätsklinikum Leipzig, Leipzig, Prof. Dr. Uwe Platzbecker; Klinikum Mannheim gGmbH, Mannheim, PD Dr.med. Stefan Klein; Universitätsklinikum Marburg GmbH, Marburg, Prof. Dr. Andreas Neubauer; Universitätsklinikum Münster, Münster, Prof. Dr. Wolfgang E. Berdel; Klinikum Nürnberg Nord, Nürnberg, Prof. Dr. Martin Wilhelm; Elblandklinikum Riesa, Riesa, Prof. Dr. Jörg Schubert; Agaplesion Diakonieklinikum Rotenburg gGmbH, Rotenburg (Wümme), Dr.med. Achim Meinhardt; Diakonie-Krankenhaus, Schwäbisch-Hall, Dr.med. Thomas Geer; Klinikum Sindelfingen-Böblingen, Sindelfingen, PD Dr.med. Markus Ritter; Robert-Bosch-Krankenhaus, Stuttgart, Prof. Dr. Walter Erich Aulitzky; HSK Wiesbaden, Wiesbaden, Dr.med. Natalia Heinz; Rems-Murr-Klinikum Winnenden, Winnenden, Prof. Dr. Markus Schaich; Universitätsklinikum Würzburg, Würzburg, Prof. Dr. Hermann Einsele

Suppl. Table 1 **Protocol allocation of patients investigated for outcome.**

Study protocol, n (%)	*UBTF*-TD^{WT} n=1404	*UBTF*-TD^{pos} n=52
AML96 (no age limit)	814 (58)	17 (33.5)
AML2003 (<=60 years)	176 (12.5)	18 (35)
AML60+ (>60 years)	42 (3)	2 (4)
SORAML (<= 60 years)	196 (14)	-
AML-Registry (no age limit)	176 (12.5)	15 (27.5)

Suppl. Table 2 **Primers used for fragment analysis and Sanger sequencing.**

UBTF E13 forward	5‘ - (6Fam) – TCCAAGAAGCCAGCCCAGGAA	chr17:44210628-44211068
UBTF E13 reverse	5‘ - AGCTGACAGCTTCCCGCCTT	

Suppl. Table 3 **Reaction mixture (A) and PCR-protocol (B) used for fragment analysis.**

(A)

**Reaction mixture (30 µl)**	
DNA (1 ng/µl)	20 µl
TBE buffer (10X)	3 µl
Forward primer (10 µM)	1.5 µl
Reverse primer (10 µM)	1.5 µl
Taq Gold polymerase	0.15 µl
dNTPs	2.4 µl
Aqua dest	1.45 µl

(B)

**PCR protocol**	
1. Denaturing	95° (11 Min)
2. Annealing	59° (1 Min)
3. Repetition (1-2)	- 31 Zyklen -
4. Elongation	72° (1 Min)
5. Final elongation	94° (30 Sec)<br>60° (40 Min)
6. Cooling	4° ∞

Suppl. Table 4 **Comutations identified via Next Generation Sequencing in 1420 *UBTF*-TD^{WT} and 52 *UBTF*-TD^{pos} patients.**

	UBTF-TD^{WT} (n=1420)	UBTF-TD^{pos} (n = 52)	p-value (adj.)
**ASXL1**			0.188
N	1290 (90.8%)	49 (94.3%)	
Y	130 (9.2%)	3 (5.7%)	
**BCOR**			0.839
N	1357 (95.6%)	50 (96.2%)	
Y	63 (4.4%)	2 (3.8%)	
**BCORL1**			0.550
N	1371 (96.5%)	51 (98.1%)	
Y	49 (3.5%)	1 (1.9%)	
**CDKN2A**			0.650
N	1418 (99.9%)	51 (98.1%)	
Y	2 (0.1%)	1 (1.9%)	
**CSF3R**			0.401
N	1401 (98.7%)	52 (100.0%)	
Y	19 (1.3%)	0 (0.0%)	
**CUX1**			0.266
N	1387 (97.7%)	52 (100.0%)	
Y	33 (2.3%)	0 (0.0%)	
**DNMT3A**			**<0.001**
N	1021 (71.9%)	51 (98.1%)	
Y	399 (28.1%)	1 (1.9%)	
**EZH2**			0.550
N	1371 (96.5%)	51 (98.1%)	
Y	49 (3.5%)	1 (1.9%)	
**GATA2**			0.061
N	1330 (93.7%)	52 (100.0%)	
Y	90 (6.3%)	0 (0.0%)	
**IDH1**			**0.027**
N	1298 (91.4%)	52 (100.0%)	
Y	122 (8.6%)	0 (0.0%)	
**IDH2**			0.317
N	1213 (85.4%)	47 (90.4%)	
Y	207 (14.6%)	5 (9.6%)	
**KDM6A**			0.524
N	1409 (99.2%)	52 (100.0%)	
Y	11 (0.8%)	0 (0.0%)	
**KIT**			0.305
N	1348 (94.9%)	51 (98.1%)	
Y	72 (5.1%)	1 (1.9%)	
**KRAS**			0.290
N	1346 (94.8%)	51 (98.1%)	
Y	74 (5.2%)	1 (1.9%)	
**NOTCH1**			0.865
N	1397 (98.4%)	51 (98.1%)	
Y	23 (1.6%)	1 (1.9%)	
**NRAS**			0.178
N	1192 (83.9%)	40 (76.9%)	
Y	228 (16.1%)	12 (23.1%)	
**PDGFRA**			0.787
N	1418 (99.9%)	52 (100.0%)	
Y	2 (0.1%)	0 (0.0%)	
**PHF6**			0.629
N	1376 (96.9%)	51 (98.1%)	
Y	44 (3.1%)	1 (1.9%)	
**PTPN11**			**0.022**
N	1321 (93.0%)	44 (84.6%)	
Y	99 (7.0%)	8 (15.4%)	
**RAD21**			0.565
N	1372 (96.6%)	51 (98.1%)	
Y	48 (3.4%)	1 (1.9%)	
**RUNX1**			0.757
N	1275 (89.8%)	46 (88.5%)	
Y	145 (10.2%)	6 (11.5%)	
**SF3B1**			0.740
N	1382 (97.3%)	51 (98.1%)	
Y	38 (2.7%)	1 (1.9%)	
**SMC1A**			0.345
N	1396 (98.3%)	52 (100.0%)	
Y	24 (1.7%)	0 (0.0%)	
**STAG2**			0.305
N	1348 (94.9%)	51 (98.1%)	
Y	72 (5.1%)	1 (1.9%)	
**TET2**			**0.007**
N	1159 (81.6%)	50 (96.2%)	
Y	261 (18.4%)	2 (3.8%)	
**TP53**			0.341
N	1316 (92.7%)	50 (96.2%)	
Y	104 (7.3%)	2 (3.8%)	
**U2AF1**			0.943
N	1377 (97.0%)	50 (96.2%)	
Y	43 (3.0%)	2 (3.8%)	
**WT1**			**<0.001**
N	1320 (93.0%)	25 (48.1%)	
Y	100 (7.0%)	27 (51.9%)	
**ZRSR2**			0.528
N	1399 (98.5%)	51 (98.1%)	
Y	21 (1.5%)	1 (1.9%)	

Suppl. Table 5 **Detailed description of clinical and cytogenetic variables in the 52 *UBTF*-TD^{pos} patients identified in this study.**

PT	Age	AML status	FAB	MDS-related changes	Karyotype	CR1	ALSCT in CR1	ALSCT SLV	DTH	RLP	Treatment
1	47	de novo	M2	NA	46, XY	Y	N	Y	Y	Y	DA; HAM; ALSCT
2	38	sAML	M2	Y	46,XY	N	N	Y	Y	N	DA; ALSCT
3	25	de novo	M6	Y	46,XY	Y	N	Y	Y	Y	DA; MAC; MAMAC; ALSCT
4	48	sAML	M4	Y	46,XY	Y	Y	N	Y	Y	MAV+MAMAC; ALSCT
5	42	de novo	M1	N	NA	N	N	Y	Y	Y	DA; ALSCT
6	46	de novo	M0	N	46,XX,+8	N	N	N	Y	N	DA
7	57	de novo	M7	Y	46,XX	N	N	Y	Y	N	DA; ALSCT
8	33	sAML	M4	Y	47,XX,+8	Y	Y	N	N	N	MAV+MAMAC; ALSCT
9	40	de novo	M1	N	46,XY	Y	N	Y	Y	Y	MAV+MAMAC; H-MAC; ALSCT
10	58	sAML	M5b	NA	46,XX	N	N	Y	N	N	DA; ALSCT
11	20	de novo	M1	N	47,XX,+8	N	N	N	Y	N	DA
12	67	MDS	RAEB	NA	47,XX,+8	Y	N	N	Y	N	I-MA1+I-MA2
13	18	de novo	M6	Y	47,XY,+8	N	N	Y	Y	Y	DA; ALSCT
14	29	de novo	M6	NA	46,XX	N	N	Y	N	N	DA; ALSCT
15	46	de novo	M6	N	46,XX,+8	N	N	Y	Y	Y	DA; ALSCT
16	45	sAML	M2	N	47,XX,+8	N	N	N	Y	N	MAV+MAMAC
17	48	de novo	M2	Y	45,X,-Y	Y	N	N	N	Y	DA MAC; autoSCT
18	20	de novo	M6	NA	46,XY,+8	N	N	Y	Y	N	DA; FLAGIDA; HiDAC; ALSCT
19	22	de novo	AML	NA	46,XX	Y	Y	Y	Y	Y	DA; ALSCT; ALSCT
20	52	sAML	M6	Y	46,XY,inv(9)(p12q12)	Y	N	N	Y	N	MAV+MAMAC
21	18	de novo	M4	Y	46,XX,der(19)t(?;19)(?;p?)	Y	Y	N	Y	Y	MAV+MAMAC; ALSCT
22	47	de novo	M2	N	47,XY,+8	Y	Y	N	Y	N	DA; ALSCT
23	40	de novo	M6	NA	NA	N	N	Y	Y	Y	DA; ALSCT
24	28	de novo	M4	Y	46,XX	N	N	Y	N	N	DA; HAM; ALSCT
25	48	sAML	M2	Y	47,XY,+8	Y	N	N	Y	Y	MAV+MAMAC; H-MAC
26	63	de novo	M1	Y	46,XY,del(12)(p12)	Y	N	Y	Y	Y	DA; FLAGIDA; ALSCT
27	35	de novo	M6	Y	46,XX	Y	N	Y	Y	Y	MAV+MAMAC; H-MAC; ALSCT
28	36	de novo	M4	NA	47, XY,+8	N	Y	N	N	N	DA; ALSCT
29	18	de novo	AML	NA	46,XX	N	N	Y	N	N	DA; FLAGIDA; ALSCT
30	17	de novo	M6	Y	47,XY,+8	N	N	Y	N	N	MAV; ALSCT
31	52	sAML	M2	N	47,XX,+8	N	N	Y	N	N	DA; HAM; ALSCT
32	30	de novo	M5	NA	47,XY,+8	Y	N	Y	Y	Y	DA; ALSCT
33	33	de novo	M5a	Y	47,XX,+8	Y	N	N	Y	Y	MAV+MAMAC; I-MAC
34	60	de novo	M2	Y	47,XY,+8	Y	N	Y	N	N	DA; MITOFLAG; ALSCT
35	49	de novo	AML	Y	46,XY	Y	N	Y	Y	Y	DA; HiDAC; MITOFLAG; ALSCT
36	22	de novo	M2	Y	46,XY	Y	N	Y	N	N	DA; ALSCT
37	47	MDS	RAEBT	NA	46,XY	Y	N	Y	N	N	DA; ALSCT
38	42	de novo	M1	N	47,XX,+8	N	N	Y	Y	Y	DA; ALSCT
39	41	de novo	M2	Y	46,XX,t(2;19),del(3q),del(13q)	Y	N	N	Y	Y	DA; Ara-C
40	35	de novo	M1	N	47,XY,+8	N	N	Y	Y	N	DA; ALSCT
41	29	de novo	M2	N	46,XY	Y	N	Y	Y	Y	MAV+MAMAC; I-MAC; ALSCT
42	77	de novo	M2	N	46,XY,del(12)(p12p13)	Y	N	N	Y	Y	I-MA
43	66	de novo	M2	N	46,XX,abn(17p)	NA	NA	NA	NA	NA	DA
44	18	de novo	M4	N	46,XY	N	N	Y	Y	Y	DA; ALSCT
45	48	de novo	M1	Y	46,XY	Y	Y	N	Y	Y	MAV+MAMAC; ALSCT
46	72	sAML	M4	NA	46,XX	Y	N	N	N	N	DA, HiDAC
47	41	de novo	M6	Y	46,XY(15)muc ish 3q26(evi 1x2),9q34(abl x2),22q11(bcr x2),11q23(mll x2)	N	N	Y	N	N	DA; ALSCT
48	22	de novo	M6	N	46,XY	Y	Y	N	Y	Y	MAV+MAMAC; ALSCT
49	51	de novo	M2	Y	46,XY	Y	Y	N	Y	Y	MAV+MAMAC; ALSCT
50	62	sAML	RAEB	Y	46,XY	Y	N	N	Y	Y	DA; MAMAC
51	19	de novo	M5	N	47,XY,+8	Y	Y	Y	Y	Y	DA; ALSCT; ALSCT
52	34	de novo	M1	Y	47,XY,+8	Y	Y	N	Y	N	MAV, MAMAC; ALSCT

Abbreviations: PT, patient (*UBTF*-TD^{pos}); CR1, first complete remission (after induction therapy); ALSCT, allogeneic hematopoietic stem cell transplantation; SLV, salvage; OS, overall survival in months; DTH, death; RFS, relapse-free survival in months; RLP, relapse; N, no; Y, yes; NA, data not available

Suppl. Table 6: **Summary of patient outcome with respect to *UBTF* mutation status in patients <50 years in multivariable analyses.**

	OS (HR)	p-val.	95%-CI	RFS (HR)	p-val.	95%-CI	EFS (HR)	p-val.	95%-CI
ELN2022 favorable risk	0.64	0.100	0.37-1.09	0.68	0.164	0.43-0.80	0.61	**0.036**	0.38-0.97
ELN2022 intermediate risk	0.97	0.902	0.56-1.67	0.94	0.839	0.68-1.28	0.94	0.798	0.59-1.51
ELN 2022 adverse risk	1.74	**0.044**	1.02-2.97	1.60	0.101	0.96-1.79	1.58	0.059	0.98-2.54
age	1.02	**0.007**	1.01-1.03	1.02	**0.017**	1.01-1.03	1.02	**<0.001**	1.01-1.03
UBTF-TD^{pos}	1.64	**0.020**	1.08-2.49	1.59	**0.039**	0.95-1.95	2.20	**<0.001**	1.52-3.17

Abbreviations: OS, overall survival; RFS, relapse-free survival; EFS event-free survival; CI, confidence interval; HR, hazard ratio

Suppl. Table 7: **Summary of patient outcome with respect to *UBTF* mutation status in multivariable analyses in the overall cohort.**

	OS (HR)	p-val.	95%-CI	RFS (HR)	p-val.	95%-CI	EFS (HR)	p-val.	95%-CI
ELN2022 favorable risk	0.57	**<0.001**	0.44-0.74	0.59	**0.001**	0.43-0.80	0.53	**<0.001**	0.42-0.67
ELN2022 intermediate risk	0.98	0.865	0.75-1.27	0.93	0.652	0.68-1.28	0.91	0.455	0.72-1.16
ELN 2022 adverse risk	1.54	**0.001**	1.21-1.97	1.31	0.085	0.96-1.79	1.58	**<0.001**	1.26-1.98
age	1.03	**<0.001**	1.02-1.03	1.02	**<0.001**	1.01-1.03	1.03	**<0.001**	1.02-1.03
UBTF-TD^{pos}	1.16	0.387	0.83-1.63	1.36	0.095	0.95-1.95	2.13	**<0.001**	1.58-1.63

Abbreviations: OS, overall survival; RFS, relapse-free survival; EFS event-free survival; CI, confidence interval; HR, hazard ratio

Suppl. Figure 1

Examples of dysplastic features in patients with *UBTF*-TDs. (A) Erythroblasts with megaloblastoid nuclei and hypogranular neutrophils, (B) granulocytes with atypically segmented nuclei and hypogranulated cytoplasm, (C) dyserythopoiesis and a hypolobulated megakaryocyte, and (D) dysmegakaryopoiesis and hypogranulated neutrophils.

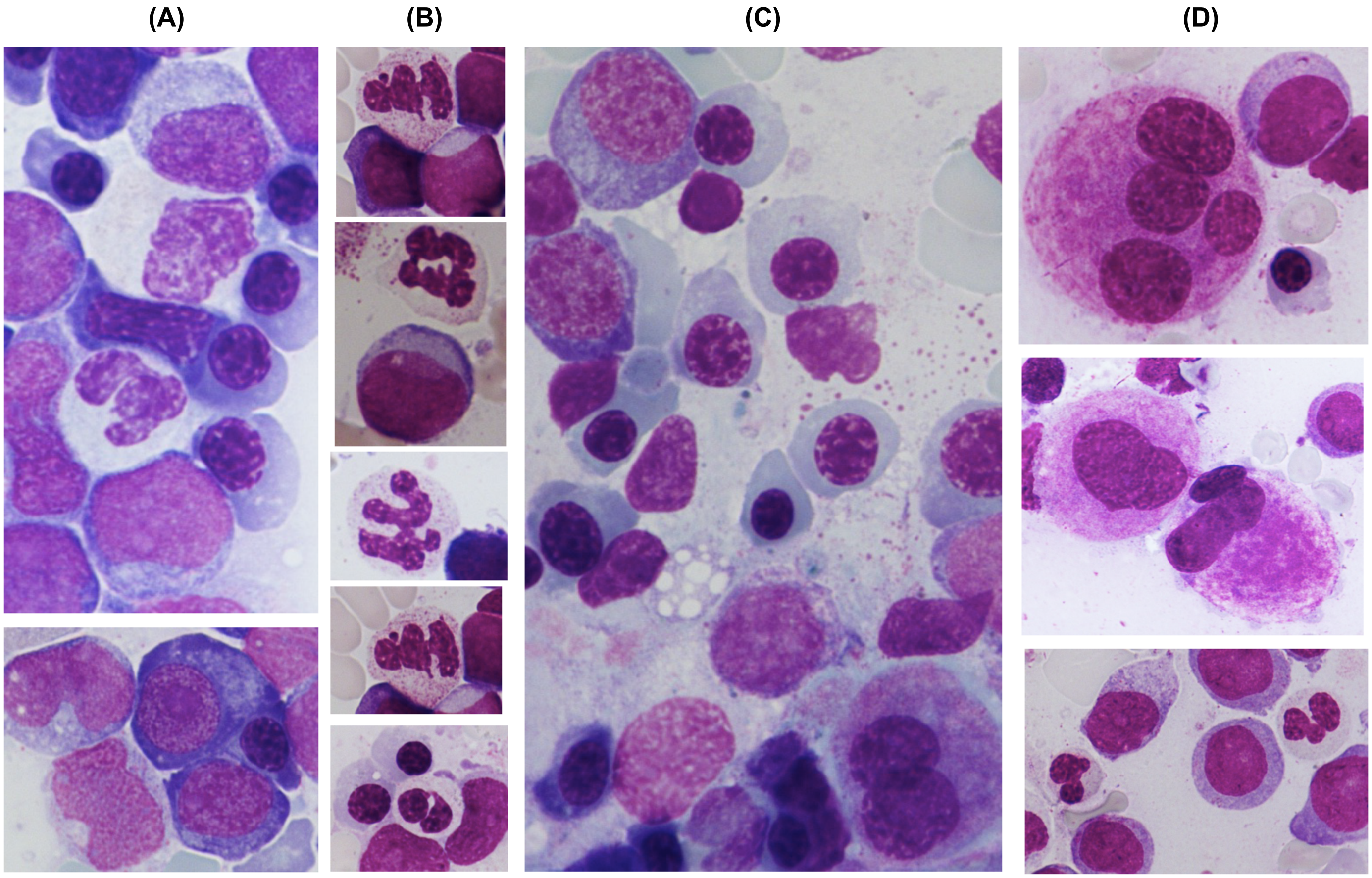

Suppl. Figure 2 Clonal evolution in *UBTF*-TD[pos] patients at relapse.

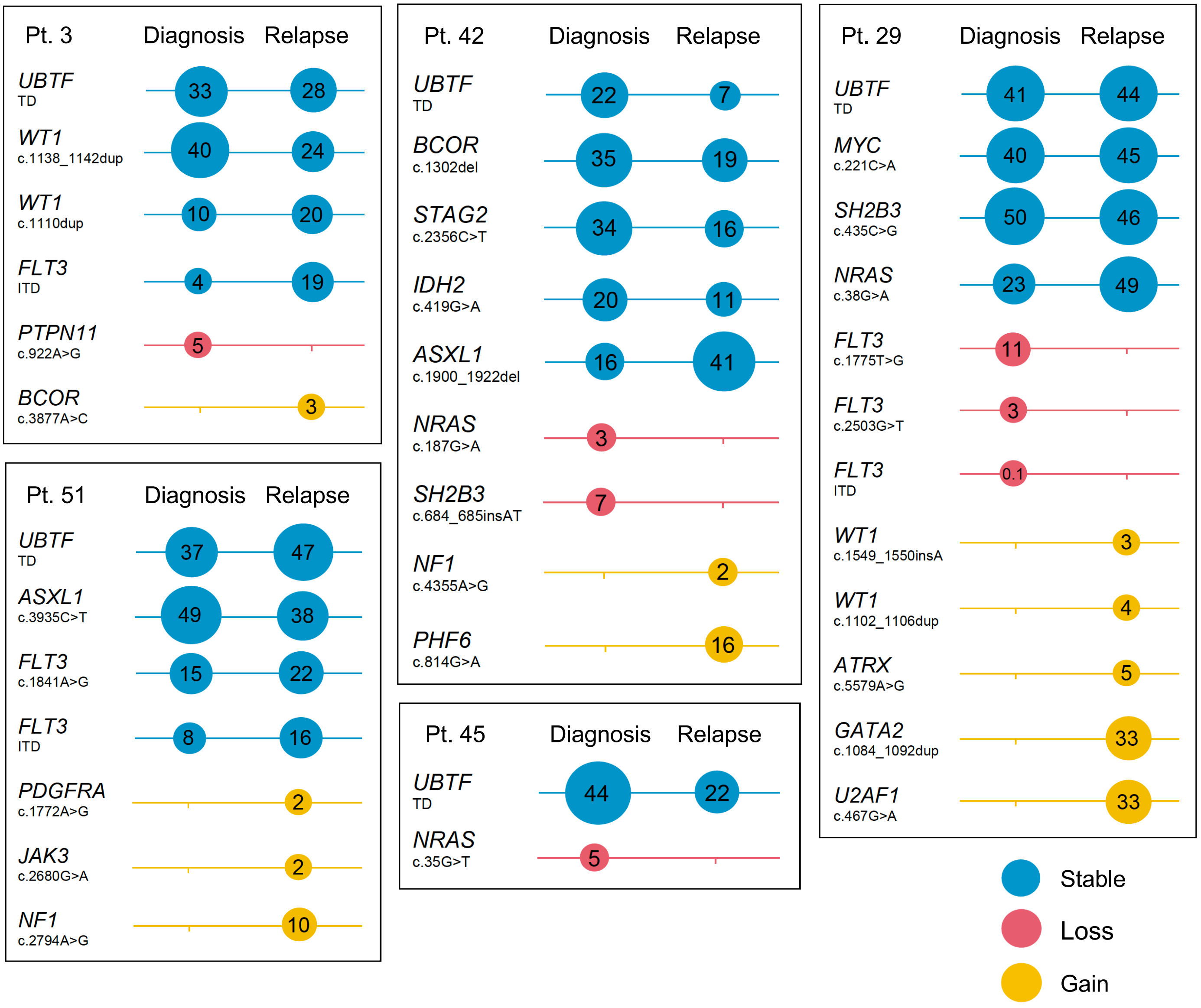

Suppl. Figure 3 **Volcano plot representation of differential expression analysis of genes in *UBTF*-TD mutant cases vs. (A) t8;21, (B) t6;9, (C) *NPM1*mut, (D) *NUP*89/*NSD1*, (E) inv16 (F) *CEBPA*$^{\text{bZIP-inf}}$.** Yellow and blue points highlight genes with significantly increased or decreased expression. X-axis shows log2-fold-changes in expression, y-axis represents log odds.

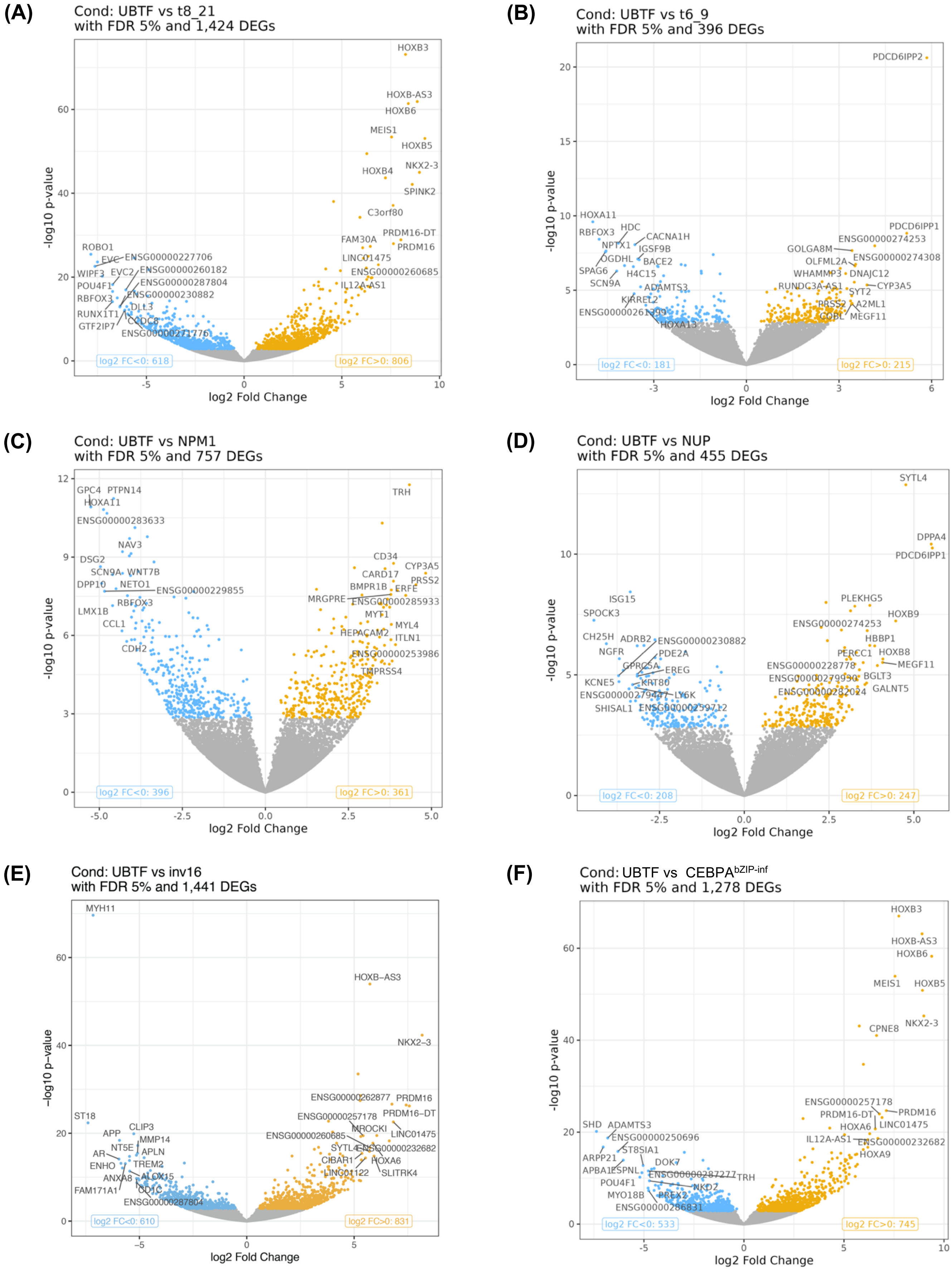

<u>Suppl. Figure 4</u> **Survival analysis according to *UBTF*-mutant status in the subgroup of ELN2022 intermediate risk patients.** Kaplan-Meier survival curves showing EFS, RFS and OS of *UBTF*-TD^{pos} and *UBTF*-TD^{WT} patients with ELN2022 intermediate risk for (A) all patients and (B) for patients <50 years of age. P-values were calculated using the log-rank test. Numbers of patients at risk are provided below the x-axis.

(A)

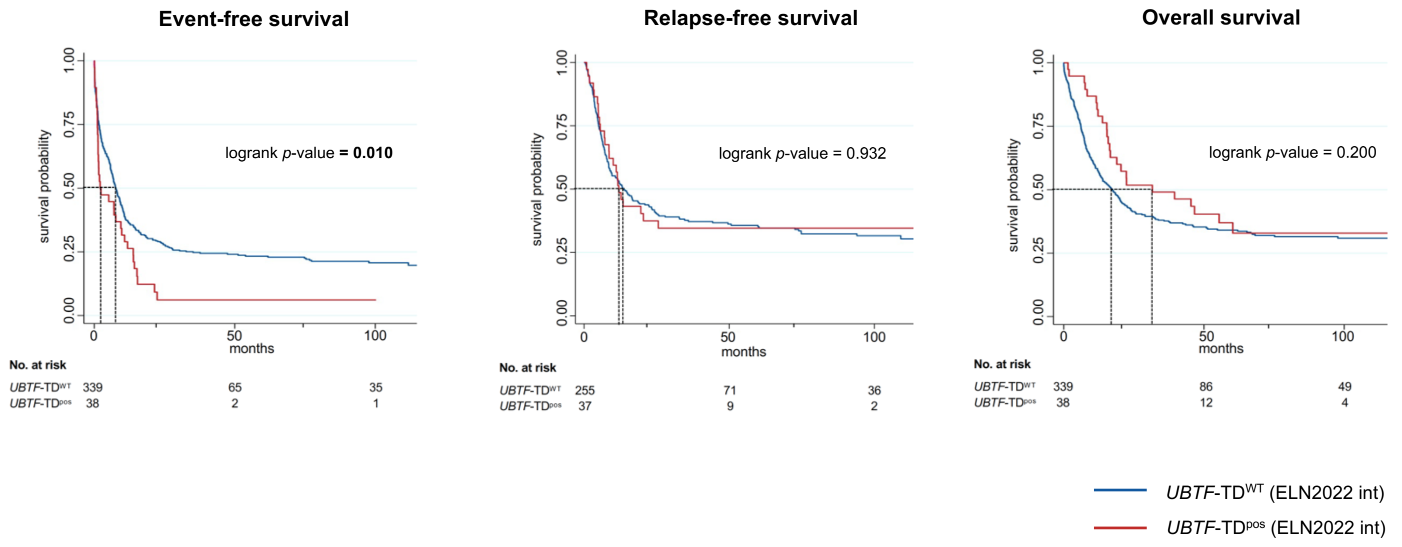

(B)

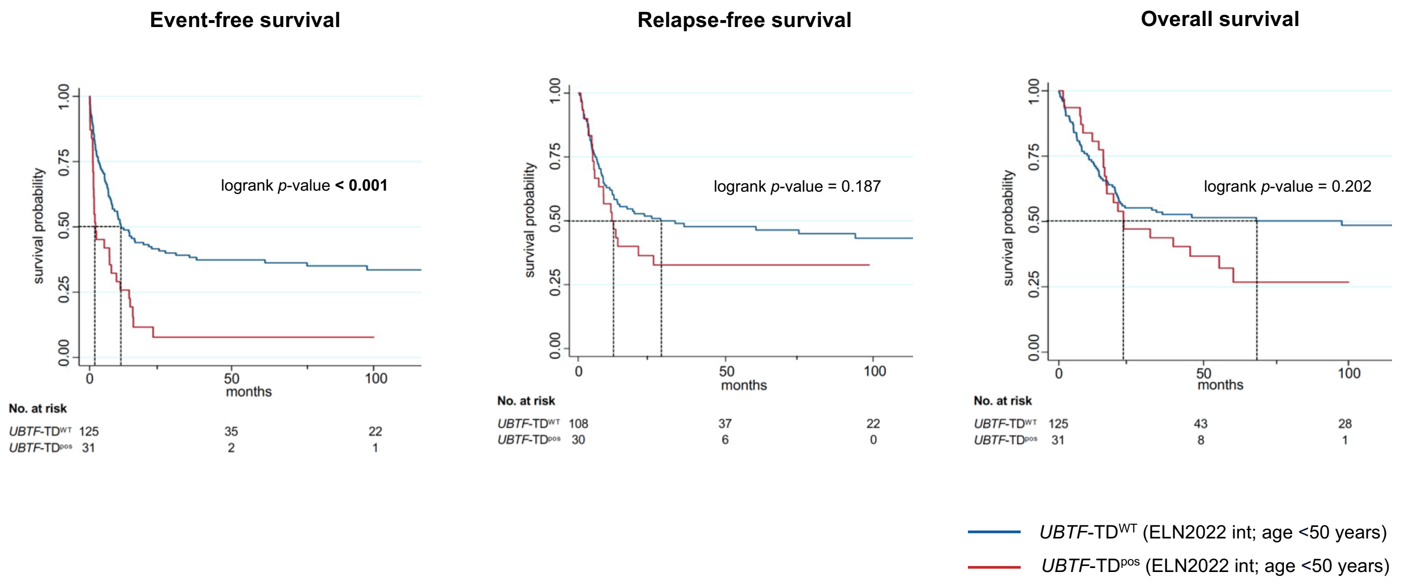

<u>Suppl. Figure 5</u> **Survival analysis according to UBTF-*TD* and (A) FLT3-*ITD* or (B) *WT1* mutant status in patients <50 years.** P-values were calculated using the log-rank test. Numbers of patients at risk are provided below the x-axis.

(A)

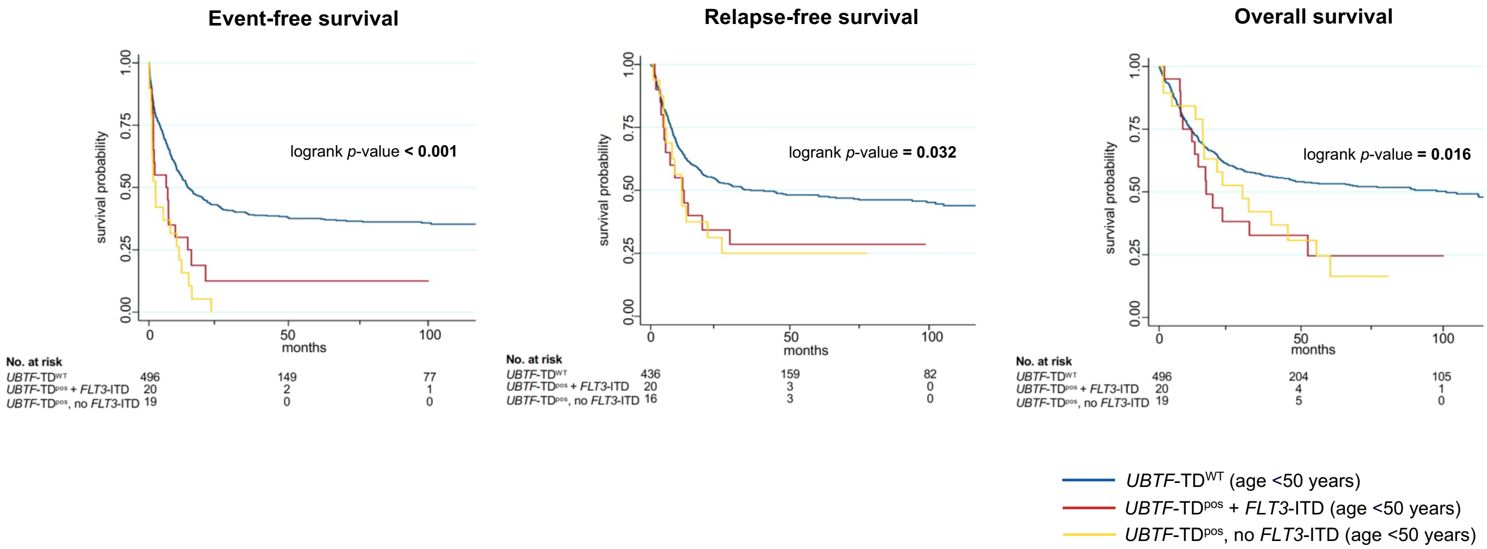

(B)

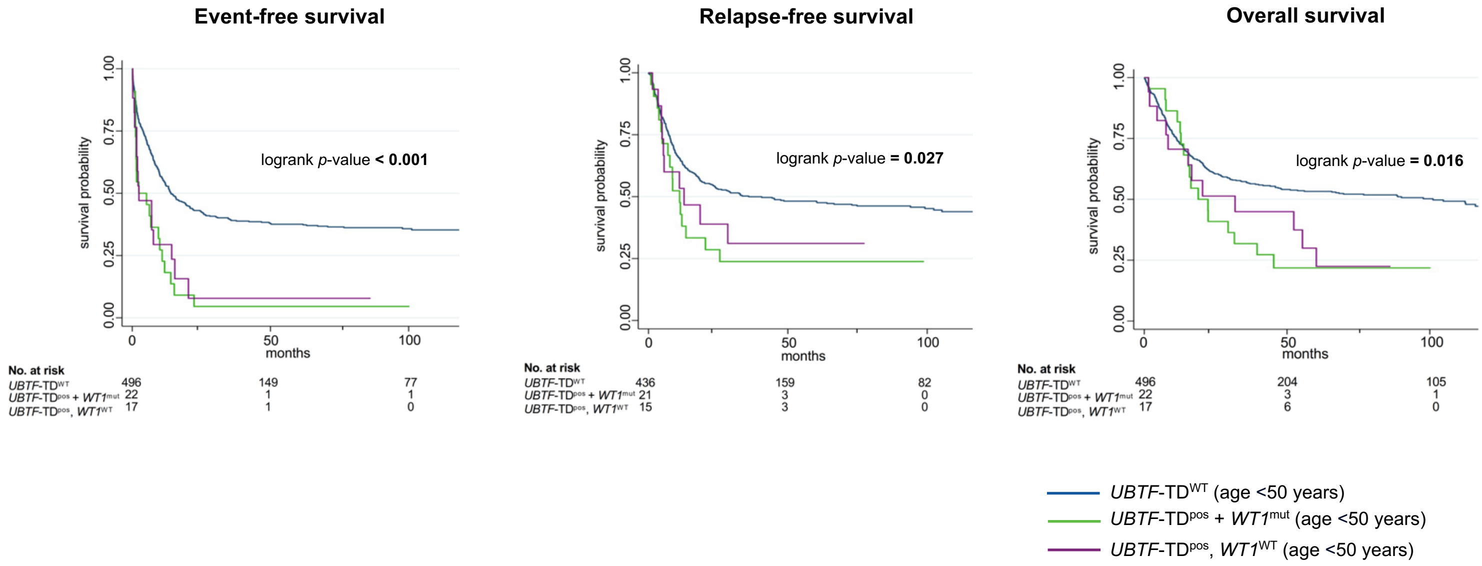

Suppl. Figure 6 **Survival analysis according to *UBTF*-TD mutational status and status of allogeneic hematopoietic stem cell transplantation in CR1**. P-values were calculated using the log-rank test. Numbers at risk are given below the x-axis.

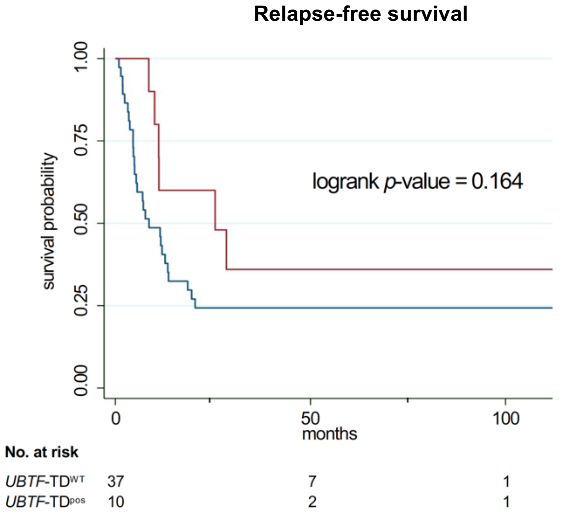

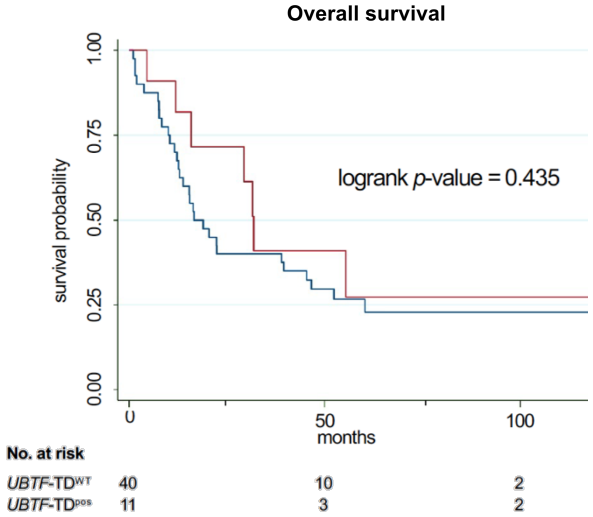

Supplement: Supplementary file 1 — Supplement [file 41408_2023_858_MOESM1_ESM.pdf]
